# Duty to treat and perceived risk of contagion during the COVID-19 pandemic: Norwegian physicians’ perspectives and experiences—a questionnaire survey

**DOI:** 10.1186/s12913-022-08905-3

**Published:** 2022-12-12

**Authors:** Karin Isaksson Rø, Morten Magelssen, Fredrik Bååthe, Ingrid Miljeteig, Berit Bringedal

**Affiliations:** 1 Institute for Studies of the Medical Profession, Oslo, Norway; 2 Centre for Medical Ethics, Institute of Health and Society, University of Oslo, Oslo, Norway; 3 Norwegian School of Theology, Religion and Society, Oslo, Norway; 4 Institute of Stress Medicine -ISM at Region VGR, Gothenburg, Sweden; 5 Institute of Health and Care Sciences, Sahlgrenska Academy, Gothenburg University, Gothenburg, Sweden; 6 Bergen Centre for Ethics and Priority Setting (BCEPS), Department of Global Public Health and Primary Care, University of Bergen, Bergen, Norway; 7 Department of Research and Development, Helse Bergen Health Trust, Bergen, Norway

**Keywords:** COVID-19, Physicians, Duty to care, Infection risk, Professional ethics

## Abstract

**Background:**

The COVID-19 pandemic actualised the dilemma of how to balance physicians´ obligation to treat patients and their own perceived risk of being infected. To discuss this in a constructive way we need empirical studies of physicians´ views of this obligation.

**Methods:**

A postal questionnaire survey was sent to a representative sample of Norwegian physicians in December 2020. We measured their perceived obligation to expose themselves to infection, when necessary, in order to provide care, concerns about being infected themselves, for spreading the virus to patients or to their families. We used descriptive statistics, chi-square tests and logistic regression analyses.

**Results:**

The response rate was 1639/2316 (70.9%), 54% women. Of doctors < 70, 60,2% (95% CI 57.7–62.7) acknowledged to some or a large degree an obligation to expose themselves to risk of infection, and 42.0% (39.5–44.5) held this view despite a scarcity of personal protective equipment (PPE). Concern about being infected oneself to some or to a large extent was reported by 42.8% (40.3–45.3), 47.8% (45.3–50.3) reported concern about spreading the virus to patients, and 63.9% (61.5–66.3) indicated worry about spreading it to their families. Being older increased the odds of feeling obligated (ExpB = 1.02 p < 0.001), while experiencing scarcity of PPE decreased the odds (ExpB = 0.74, p = 0.01). The odds of concern about spreading virus to one´s family decreased with higher age (Exp B = 0.97, p < 0.001), increased with being female (Exp B = 1.44, p = 0.004), and perceived lack of PPE (Exp B = 2.25, p < 0.001). Although more physicians working in COVID-exposed specialties experienced scarcity of PPE and reported perceived increased risks for health personnel, the odds of concern about being infected themselves or spreading the virus to their families were not higher than for other doctors.

**Conclusion:**

These empirical findings lead to the question if fewer physicians in the future will consider the duty to treat their top priority. This underscores the need to revisit and revitalise existing ethical codes to handle the dilemma between physicians´ duty to treat versus the duty to protect physicians and their families. This is important for the ability to provide good care for the patient and the provider in a future pandemic situation.

## Background

The first phase of the COVID-19 pandemic actualised the dilemma of physicians' and other health personnel's duty to treat patients despite their own risk of being infected with a serious, potentially lethal, disease.

With increasing knowledge, effective treatment, and the use of personal protective equipment (PPE), the risks of occupationally contracted infections have been substantially reduced for physicians, especially in high-income countries. In Norway, tuberculosis and blood-borne viruses, the most common occupationally contracted infections in recent years, were registered in only 20–40 health personnel during a 15-year period (1992–2017), and only a few of these concerned physicians [1].

The start of the COVID-19 pandemic in the spring of 2020 was a threatening scenario for both patients and healthcare workers, with potentially severe consequences, as seen for example in Italy [2]. Various mobilising strategies were rapidly implemented throughout the health care system in Norway [3]. The reallocation of personnel and preparing healthcare for the potential wave of infected patients involved placing some physicians in settings with previously unfamiliar types of patients [4]. In addition to the capacity limitations, there was a lack of effective personal protective equipment (PPE) in both hospitals and primary/municipal care, that is general practice, nursing homes for elderly and home care [3, 5]. The scarcity of PPE combined with the treatment of patients infected with the new COVID-19 virus, created a situation with an increased risk of serious infection for health care providers. Indeed, studies have confirmed that Norwegian health providers experienced an increased risk of being infected by COVID-19 in 2020, especially in the first wave of the pandemic (February 2020- July 2020), and the lack of PPE was probably a contributing factor [6].

Do physicians and other health professionals have a duty or an obligation to treat patients when this exposes them to the risk of contagion? The scope and conditions of such a duty have been discussed in the literature [7–18].

On the one hand, the social contract between the profession and society underscores the duty to treat patients, which includes loyalty to colleagues and to health institutions to give epidemic patients the treatment they need [7]. This duty to treat is enshrined in national medical ethical codes and in the World Medical Organization's Geneva Declaration. For instance, during the Spanish flu in 1918, the American Medical Association's (AMA´s) code of medical ethics explicitly stated that “physicians were expected to continue their provision of care to patients without regard to the risk to [their] own health”[7]. In their discussion of physician's moral character and virtues, Pellegrino and Thomasma argued that physicians should exhibit the virtue of ‘self-effacement’ [16]. In times of pandemics, this can imply the acceptance of a certain risk of infection.

On the other hand, physicians also have a duty to take care of themselves [19], and they have obligations to others, such as family members, colleagues, and patients suffering from other diseases [7, 13, 20]. Furthermore, the employer or institution has an obligation to mitigate risks, for instance by supplying adequate PPE [14].

Although physicians´ obligation to treat has been discussed in the literature, few empirical studies have investigated their perspectives [11, 12]. Against this background, our research group wanted to examine physicians´ views of their obligation to treat patients despite the risk of contagion, and how they experienced concerns for themselves and others regarding the risk of infection.

## Materials and methods

### Aim, design and setting of the study

We examined the views of physicians working in Norway regarding their obligation to treat patients despite the risk of contagion and how they experienced concerns for themselves and others regarding the risk of infection. We examined how age, gender, work in different parts of the healthcare system, and access to PPE related to their views and concerns.

### Participants

In December 2020, a postal questionnaire survey was sent to 2316 members of a panel of actively working physicians in Norway (the Norwegian Physician Survey). The panel was established in 1994 and is surveyed every second year. A randomly selected group of 2000 doctors was originally invited to participate in a longitudinal study, and 1272 (64%) agreed. Approximately every four years, a randomly selected sample of the youngest doctors are invited to join the panel, replacing respondents who have left, due to retirement, death or voluntary withdrawal [21]. The sample is representative of physicians working in Norway regarding gender, age and place of work; see Table 1. Place of work was defined by grouping specialties in relation to COVID-19-exposion. In the comparison of the proportion of specialists, we used the numbers for certified specialists among Norwegian doctors in general, while the proportions in the sample from this study include both certified specialists and specialists in training for the relevant specialty. Since the number of specialists in training should be related to the number of specialists in each specialty, we assume that these proportions are comparable. Statistics for Norwegian doctors in 2021 were obtained from the Norwegian Medical Association (www.legeforeningen.no/om-oss/legestatistikk/om-leger-i-norge/ accessed 20 September, 2022).

**Table 1:** Gender, age and place of work among respondents (< 70) compared to doctors working in Norway (< 70)

	Respondents in the present study (< 70 years)	Doctors working in Norway 2021 (< 70 years)
Gender, women (%, 95%CI)	56.0% (862/1540)	54.4% ^1^
Average age (years, SD)	44.3 (SD 12.1) (N = 1540)	43.9 (SD 11.4) ^1^
Place of work		
Doctors working in "COVID-exposed specialties" (%, 95%CI)	8,3% (123/1487)	9.1% (1528/16851) ^2^
Doctors working in other somatic hospital specialties (%, 95%CI)	42.0% (624/1487)	43.1% (7257/16851) ^2^
Doctors working as General practitioners (%, 95%CI	22,1% (329/1487)	23.9% (4031/16851) ^2^
Doctors working in psychiatry/ laboratory/diagnostic medicine (%, 95%CI)	27,6% (411/1487)	23.9% (4035/16851) ^2^

^1^ N for all doctors < 70 years in Norway = 30,360

^2^ N for certified specialists < 70 years = 16,851

### Main outcome measures

The main outcome measures were the perceived obligation to expose oneself to infection in general and under a scarcity of personal protective equipment (PPE) when necessary to provide care. We also studied perceived concerns about spreading the virus to patients, being infected and spreading the virus to one´s family. Response alternatives were given on a four-point Likert scale where agreement to each statement was scored from "not at all" to "to a large degree" or "not relevant for me" (See questionnaire items in Table 2). For the analyses the response alternatives were dichotomised into scores of “not at all” and “to a small degree” (0) versus “to some degree” and “to a large degree” (1), while those who scored “not relevant for me” were excluded.

**Table 2:** Perceived obligation to provide care as well as concerns about the contagion and scarcity of PPE. (Doctors < 70 years old)

Main outcome measures		Not at all (%, 95%CI)	To a small degree (%, 95%CI)	To some degree (%, 95%CI)	To a large degree (%, 95%CI)	Not relevant for me (%, 95%CI)	N
Questions concerning the whole pandemic period thus far, March 2020 to December 2020	Do you agree that physicians have an obligation to expose themselves to the risk of infection when necessary to provide healthcare?	11.9 (10.3–13.5)	25.9 (23.7–28.1)	46.5 (44.0–49.0)	13.7 (12.0–15.4)	2.0 (1.3–2.7)	1508
	Do you agree that physicians have an obligation to provide healthcare even if the employer cannot provide sufficient PPE	25.5 (23.3–27.7)	29.3 (27.0–31.6)	34.4 (32.0–36.8)	7.6 (6.3–8.9)	3.1 (2.2–4.0)	1504
	Have you been concerned about infecting patients with COVID-19?	15.7 (13.9–17.5)	32.4 (30.0–34.8)	29.4 (27.1–31.7)	13.4 (11.7–15.1)	9.0 (7.6–10.4)	1505
	Have you been concerned about being infected yourself with COVID-19?	11.5 (9.9–13.1)	38.5 (36,0–41.0)	35.3 (32.9–37.7)	12.5 (10.8–14.2)	2.3 (1.5–3.1)	1508
	Have you been concerned about infecting your family members with COVID-19?	7.4 (6.1–8.7)	25.9 (23.7–28.1)	35.7 (33.3–38.1)	28.5 (26.2–30.8)	2.5 (1.7–3.3)	1508
Scarcity of PPE							
Questions concerning the first part of the pandemic (March to May 2020)	To what degree did you experience a scarcity of PPE?	11.8 (10.2–13.4)	22.6 (20.5–24.7)	32.6 (30.2–35.0)	22.2 (20.1–24.3)	10.8 (9.2–12.4)	1505
	To what degree did you experience that a scarcity of PPE led to sub-optimal treatment of patients?	34.4 (32.0–36.8)	30.4 (28.1–32.7)	16.6 (14.7–18.5)	4.6 (3.5–5.7)	14.7 (12.9–16.5)	1505
	To what degree did you experience that a scarcity of PPE led to increased risk for patients?	34.8 (32,4–37,2)	30.1 (27.8–32.4)	16.4 (14.5–18.3)	4.4 (3.4–5.4)	14.1 (12.3–15.8)	1505
	To what degree did you experience that a scarcity of PPE led to increased risk for personnel?	24.4 (22.2–26.6)	25.4 (23.7–28.1)	24.7 (23.2–26.6)	13.3 (11.6–15.0)	12.2 (10.5–13.9)	1505

### Other variables

Gender was defined as male or female and age was reported as a continuous variable. In the results section we have generally included doctors < 70 years to focus on those who are in full clinical practice.

We measured the perceived scarcity of PPE and the perceived consequences for patients and personnel of such scarcity during the first part of the pandemic (March to May 2020). See the questionnaire items in Table 1. Response alternatives were given on a four-point Likert scale where agreement to each statement was scored from "not at all" to "to a large degree" or "not relevant for me". For the analyses the response alternatives were dichotomised into scores of “not at all” and “to a small degree” (0) versus “to some degree” and “to a large degree” (1), while those who scored “not relevant for me” were excluded. Place of work*:* We grouped physicians into four places of work, hypothesising that different groups of specialties would undergo different kinds of challenges during such pandemics. (i) Emergency medicine, anaesthesiology, infectious medicine and pulmonary medicine are described here as "COVID-19-exposed specialties". There were two other groups with direct somatic contact with patients (ii) other somatic hospital specialties (including internal medicine specialties, paediatrics, neurology, rheumatology, oncology, dermatology, surgical specialties, gynaecology, ears- nose and throat, ophthalmology) and (iii) general practitioners and others in primary care. The last group (iv) included psychiatry/laboratory/diagnostic medicine (psychiatry, occupational medicine, social medicine, addiction medicine, clinical pharmacology, clinical neurophysiology, clinical biochemistry, medical genetics, medical microbiology, nuclear medicine, pathology, radiology and doctors who responded "none of these"– like researchers, or on maternity leave). Both senior and junior physicians working in their respective specialties were included [21] (See Table 1). In the regression analyses "other somatic hospital specialties" was used as the reference group (since this group includes most respondents). In turn the other specialty groups are compared to this reference.

### Statistics

The sample is presented with descriptive data using the number of respondents, proportions (%) and mean values. Differences between proportions were assessed with chi-square tests. Differences between mean values were tested with independent t-tests.

The associations between age, gender, place of work, and perceived scarcity of PPE with the outcome variables were investigated with univariate and multivariate logistic regression analyses. SPSS Version 19 was used. The significance level was set to < 0.05. Missing values were omitted from each analysis.

## Results

The response rate was 1639/2316 (70.9%). The sample is described in Table 1 and compared to working doctors in Norway. Of doctors < 70 years, 56.0% were women. The average age for women was significantly lower than for men: 41.7 years (SD 10.9) vs 47.7 years (SD 12.8); t = 10.6, *p* < 0.001. The distribution of physicians regarding their place of work is shown in Table 1.

### Perceived obligation to expose oneself to the risk of infection to treat patients and concerns about spreading and being infected by COVID-19

Of doctors < 70 years of age a majority,60.2% (95% CI 57.7–62.7) acknowledged an obligation to some or a large degree to expose themselves to the risk of infection to treat patients (see Table 2). This number was reduced to 42.0% (95% CI 39.5–44.5) if their employer could not offer adequate PPE.

42.8% (95% CI 40,3–45,3)were to some or a large degree worried about spreading the virus to patients. In addition, 47.8% (95% CI 45.3–50.3) were concerned about being infected themselves, and 63.9% (95% CI 61.5–66.3) were worried about spreading the virus to their families. See Table 2.

### Scarcity of PPE and consequences for patient treatment and personnel

For respondents < 70 years of age 54.8% (95% CI 52.3–57.3) experienced scarcity of PPE to some or a large degree in the first phase of the pandemic, while 21.2% (95% CI 19.1–23.3) considered this to have led to poorer care (Table 2). Moreover, 20.8% (95% CI 18.7–22.9) reported that scarcity led to increased risk for patients, and 38.0% (95%CI 35.5–40.4) reported that the risk for health personnel increased. We performed chi-square tests to compare physicians working in different specialties for each of these items. When excluding those who answered "not relevant for me", physicians working in COVID-exposed specialties were significantly more likely than physicians from other specialties to experience a scarcity of PPE to some or a large degree (78.2% vs 61.6% for other somatic hospital specialties, 60.6% for GPs and 56.3% for psychiatry/ laboratory/diagnostic medicine; chi-square test 17.6, p < 0.001, df = 3). Physicians working in COVID-exposed specialties were also more likely to perceive that scarcity led to poorer care for patients (37.3% vs 20.8%, 29.4% and 19.6% respectively for the other groups; chi-square test 22.1, p < 0.001, df = 3), that scarcity led to increased risks for patients (33.1% vs 21.4%, 29.0% and 23.9% respectively for the other groups, chi-square test 11.3, p = 0.01, df = 3) and that scarcity led to increased risk for personnel (60.5% vs 42.7%, 40.9% and 39.6% respectively for the other groups; chi-square test 16.7, p < 0.001, df = 3).

### Associations between outcome variables and age, gender, perceived PPE scarcity and workplace

Using univariate and multivariate logistic regression analyses the associations were examined between each outcome variable and age, gender, perceived scarcity of PPE and workplace. The multivariate results are reported in Table 3.

**Table 3:** Multivariate associations for physicians < 70 years between each outcome variable A-E (see note under table) and age, gender, perceived scarcity of PPE and workplace

		**A**	**B**	**C**	**D**	**E**
		Exp(B) (95% CI) N = 1318	Exp(B) (95% CI) N = 1296	Exp(B) (95% CI) N = 1265	Exp(B) (95% CI) N = 1316	Exp(B) (95% CI) N = 1314
	Constant	0.71	0.27***	1.55	0.76	8.34***
	Age	1.02*** (1.01–1.03)	1.03*** (1.02–1.04)	0.97*** (0.96–0.98)	1.00 (0.99–1.01)	0.97*** (0.96–0.98)
	Gender (male = 0, female = 1)	0.98 (0.77–1.24)	0.99 (0.79–1.25)	1.61*** (1.27–2.04)	1.16 (0.92–1.46)	1.44** (1.13–1.85)
	Perceived scarcity of PPE (not at all/to a small degree = 0, to some/a large degree = 1)	0.74* (0.58–0.93)	0.99 (0.79–1.25)	1.53*** (1.21–1.93)	1.92*** (1.53–2.42)	2.25*** (1.77–2.88)
	Workplace – reference category "other somatic hospital specialties"					
	Workplace (COVID-19-exposed specialties = 1 vs other somatic = 0)	0.89 (0.59–1.33)	0.94 (0.62–1.41)	0.94 (0.62–1.42)	0.69 (0.46–1.04)	0.61* (0.39–0.94)
	Workplace (psychiatry/ laboratory/diagnostic medicine = 1 vs other somatic = 0)	0.99 (0.74–1.32)	1.03 (0.77–1.37)	0.87 (0.64–1.17)	0.96 (0.72–1.27)	0.68* (0.50–0.92)
	Workplace (general practice = 1 vs other somatic = 0)	0.99 (0.74–1.32)	0.88 (0.66–1.17)	1.27 (0.95–1.70)	1.13 (0.85–1.49)	0.71* (0.52–0.96)

^*^ < 0.05 ** < 0.01 *** < 0.001

**A**. Associations regarding the perceived obligation to expose oneself to the risk of infection when necessary to provide healthcare. R^2^ = 7.4 (Hosmer&Lemeshow), .024 (Cox&Snell), .033 (Negelkerke). Model χ^2^ = 32.1

**B**. Associations regarding the perceived obligation to provide healthcare even if the employer cannot provide sufficient PPE. R^2^ = 9.6 (Hosmer&Lemeshow), .026 (Cox&Snell), .035 (Negelkerke). Model χ^2^ = 34.1

**C**. Associations regarding concern for infecting patients with COVID-19. R^2^ = 7.5 (Hosmer&Lemeshow), .055 (Cox&Snell), .074 (Negelkerke). Model χ^2^ = 71.8

**D**. Associations regarding concern for being infected with COVID-19. R^2^ = 17.3 (Hosmer&Lemeshow), .030 (Cox&Snell), .040 (Negelkerke). Model χ^2^ = 39.9

**E**. Associations regarding concern for infecting one´s family with COVID-19. R^2^ = 2.4 (Hosmer&Lemeshow), .093 (Cox&Snell), .129 (Negelkerke). Model χ^2^ = 128.1

The odds of agreeing to some or to a large degree with an obligation to expose oneself to the risk of infection when necessary to provide healthcare, in univariate analyses, was significantly associated with male gender, higher age and scarcity of PPE. The association with gender disappeared in the multivariate analyses, where we found that the odds increased significantly with higher age (Exp B = 1.02, p < 0.001) and decreased for doctors who had experienced scarcity of PPE (Exp B = 0.74, p = 0.011), controlled for gender and workplace. See Table 3A.

The odds of reporting a perceived obligation to provide healthcare even if the employer cannot provide sufficient PPE to some or to a large degree was, in univariate analyses, significantly associated with higher age and with male gender. The association with gender disappeared in the multivariate analyses, where higher age (Exp B = 1.03, p < 0.001) remained the only significant association, controlled for gender, workplace and perceived scarcity of PPE. See Table 3B.

The odds of being concerned with infecting patients with COVID-19 to some or to a large degree was, in univariate analyses, associated with age, gender and scarcity of PPE. These associations remained in the multivariate model where the odds for concern decreased with higher age (Exp B = 0.97, p < 0.001), increased with being female doctor (Exp B = 1.61, p < 0.001), and increased when having experienced a scarcity of PPE (Exp B = 1.53, p < 0.001), controlled for work place. See Table 3C.

The odds of being concerned about being infected with COVID 19 oneself to some or a large degree was, in univariate analyses, significantly associated with being a woman and with perceived scarcity of PPE. In multivariate analysis the association with gender disappeared, and perceived scarcity of PPE (Exp B = 1.92, p < 0.001) remained a significant factor, where more scarcity was associated with more concern, controlled for age, gender and work place. See Table 3D.

The odds of being concerned about infecting one´s family with COVID 19 to some or to a large degree was, in univariate analyses, significantly associated with gender, age, perceived lack of PPE and with work place. These associations remained in the multivariate analyses, where the odds of being concerned decreased with higher age (Exp B = 0.97, p < 0.001), increased with being female doctor (Exp B = 1.44, p = 0.004), and increased with a perceived lack of PPE (Exp B = 2.25, p < 0.001). Working in COVID-exposed specialties (Exp B = 0.61, p = 0.025), in psychiatry/ laboratory/diagnostic medicine (Exp B = 0.68, p = 0.014) and in general practice (Exp B = 0.71, p = 0.025) significantly decreased the odds for such concern compared to working in other somatic hospital specialties. See Table 3E.

## Discussion

### Main findings

Most (60%) of the surveyed physicians, acknowledged an obligation to become exposed to COVID-19 infection to provide care to some or a large degree. When the physicians experienced a scarcity of PPE, 42% agreed. The odds of acknowledging these obligations increased with age.

Almost half of the respondents were to some or to a large extent concerned about being infected by COVID-19 themselves, and about spreading the virus to their patients. Almost 64% worried about spreading the virus to their families. The odds of concern regarding spreading the virus to patients or family increased with younger age and with female gender. A scarcity of PPE was experienced by 55% of the respondents, and experiencing scarcity decreased the odds of acknowledging obligation to treat, and increased the odds of concern about contagion.

### Perception about duty to treat

In this study, 60% of the physicians acknowledged a duty to treat, despite risking contagion. Because the survey was sent during a pandemic, many respondents had recent experiences to draw on when they answered. Other studies have used hypothetical scenarios. In a study from 2003 in the US, a lower percentage—about half of the physicians—reported a duty to treat in a hypothetical outbreak of a potentially deadly illness [11]. In a 2006 survey of employees in a German university hospital, 24% of physicians agreed that it was ethical to refrain from providing care; thus 76% thought there was a predominant duty to treat during a hypothetical influenza pandemic [12].

### Balancing the duty to treat and the duty to protect healthcare workers and their families

Conflicts between physicians´ duties have been discussed concerning previously threatening epidemics or pandemics, such as those caused by HIV/AIDS (Human Immuno-deficiency Virus/Acquired Immuno Deficiency Syndrome), Ebola or SARS (Severe Acute Respiratory Syndrome) [7, 8, 10]. On the one hand, there is concern that the duty to treat patients during epidemic or pandemic outbreaks has eroded [10]. On the other hand, there is an increasing focus on the duty to care for carers´ own health, which is both intrinsically important and instrumentally necessary for them to provide their patients with good care in the longer term [19, 22].

A US study found the concern for one´s family safety to be the most significant barrier that would prevent health personnel (including physicians) from signing in to work in an influenza pandemic [23]. In a German survey, about a quarter of physicians found it ethical to refrain from providing care to protect themselves and their families [12]. Among the Norwegian physicians in this study, we found a difference between older and younger physicians. The odds were significantly higher for older physicians to report the traditional obligation or "duty to treat". Younger physicians and female physicians had higher odds of experiencing concern about spreading the virus to patients and to their own families.

These distinctions between age groups and genders could be stable between generations (due to life phases and professional experience). Conversely, it might also result from sociocultural changes in medicine, as in society in general. If the traditional medical duty to treat to a larger degree becomes challenged by the duty to protect healthcare workers and their families from infection, this can imply, over time, that fewer physicians will consider the duty to treat their top priority.

### Do physicians from all specialties feel equally obligated?

Physicians working in "COVID-19-exposed specialties" had significantly lower odds of concern about spreading the virus to their families. These physicians did not have higher odds of concern about being infected themselves, even though they actually experienced more scarcity of PPE than others and reported that this led to higher risks for health personnel. Thus, one could surmise that these physicians were less stressed by the situation or that they were more accepting of these kinds of risks. However, physicians in these specialties do not acknowledge a duty to treat patients more often than others.

The findings are important regarding whether certain specialties or groups of physicians should have a stronger duty to treat than others. Malm et al. question the extent to which physicians, in general, feel bounded by the relevant provisions in ethical codes or the Geneva Declaration [8]. There can be an implied consent to abide by such general codes when starting one’s career as a physician. However, the authors claim that many physicians will not have internalised and thought through what this means in different scenarios. They further argue that one might establish special contracts or codes detailing a special duty to treat for physicians who work in specialties that are more regularly exposed to such risks and, subsequently, have more training in handling them. One example of such experience and training could be treating contagious patients in departments for infectious diseases [8].

### PPE and the employers´ duties

Our study has demonstrated a link between scarcity of PPE and less support for the duty to treat. The scarcity of PPE is also linked to more concern about the infection of self and others. Cowper et al. emphasise the potential tension between the employer's dual duties in a pandemic situation: to provide adequate treatment for patients and to provide physicians and other employees with a safe work situation, in this case, by providing adequate PPE [14]. The responsibility to provide healthcare workers with PPE can be the employer´s, but Schuklenk emphasises that governments are also responsible for securing an adequate stock of PPE. This is especially the case when a pandemic has been anticipated, as was the case with COVID-19 [15]. Thus, insufficient pandemic preparedness on the part of the governments and institutions arguably weakens physicians’ moral obligation to care. Johnson and Butcher argue that physicians who make sacrifices to provide care during a pandemic are owed reciprocal obligations from their institutions and society [17]. The provision of adequate PPE could be such an obligation. The authors claim that opting out of a high-risk procedure because adequate PPE is unavailable could be justified.

Britain´s General Medical Council has acknowledged this dual duty in relation to the present pandemic: "We do not expect physicians to leave patients without treatment, but we also don´t expect them to provide care without regard to the risks to themselves or others" [9]. In the Norwegian Medical Association's ethical code for physicians, the obligation to treat patients is emphasised, but nothing is stated about how this obligation should be balanced against potential risks to physicians´ own health [24]. A recent paper by Gamlund et al. argues that neither the Declaration of Geneva, the Norwegian Medical Association's ethical guidelines, nor Norwegian laws give a precise answer to whether physicians have a moral obligation to provide medical care, without adequate PPE, during a pandemic [18].

### Frameworks for balancing duties

The fact that about 40% of the physicians in this study, and a substantial number of physicians in previous studies [11, 12], are hesitant to expose themselves to the risk of infection, underscores the importance of finding ways to handle this dilemma. Different frameworks have been proposed for balancing duties and interests that might conflict.

McDougall et al. [20] describe a structure for individual reflection, staff discussions and decision making. They stress the importance of transparency and accountability for the decisions made. Ethically challenging dilemmas must be verbalised and discussed, and healthcare workers must understand and participate in deliberations. Participation and knowledge will increase the acceptance of difficult decisions.

McConnell developed a deontological framework (defining actions that are good or bad according to a clear set of rules) for evaluating health professionals’ duty to provide care [13]. He argues that professionals are morally justified to refrain from care when their duty to treat is outweighed by the risks and burdens to themselves. The obligation to protect family members can be a significant part of such burdens. He suggests that healthcare workers exposed to a higher risk of infection should be compensated, for example with prioritised healthcare for their family. Meanwhile, a healthcare worker relocated from a higher risk situation might be morally required to compensate society for this, for example financially or with free labour after the epidemic. Deontological frameworks like McConnells could inspire to thorough and broad normative discussions and deliberation processes with relevant stakeholders. Our study can be a useful empirical contribution into these processes.

### Strengths and weaknesses

A strength of this study is the closeness in time between the start of the COVID-19 pandemic and the measurements of physicians´ attitudes and concerns pertaining to this period. This can contribute to results that are closer to the practical dilemmas experienced in healthcare during a pandemic and not just measure theoretical ideals of what physicians´ duties to patients entail.

The relatively high response rate (70%), which is higher than for other surveys of the medical profession [25], and the fact that the sample is representative of practising physicians in Norway in key aspects [21], provide a good basis for generalisation to the population of doctors. There were also no significant disparities regarding age or gender between the respondents and the non-respondents. This does not, however, rule out the possibility of nonresponse bias.

The pandemic laid great stress on large parts of the healthcare system, particularly its personnel. There is a possibility that the most stressed physicians opted out of answering the questionnaire. Conversely, the most burdened physicians might have wished to document the situation in which they found themselves. Although the scarcity of PPE and perceived risk were high in the first months of the pandemic in Norway, as in other countries, it soon improved. Compared to other countries, the infection rates, hospitalisation and lack of resources have been low. Therefore, the results in this study could be difficult to generalise to other countries.

Because attitudes and concerns can also vary with personality and coping style [26] it could be important to include such co-variates in future analyses. Another limitation could be that we have only self-reported data in this study. However, when investigating attitudes and concerns, this is a plausible method.

We did not used validated questions in this study, as we could not find good examples that explored what we wanted to study.

## Conclusion

Our study points to the challenging dilemma that the COVID-19 pandemic has actualised. If the traditional medical duty to treat to a larger degree becomes challenged by the duty to protect healthcare workers and their families from infection, this can imply, over time, that fewer physicians will consider the duty to treat their top priority.

As healthcare and society are in transition, national and international ethical codes (including the Geneva Declaration) no longer seem sufficient to handle this dilemma. Additional ethical frameworks have been proposed that increase transparency and involve physicians and other health personnel in decision making, as well as explicitly differentiating the "duty to treat" between specialties or individuals. Research concerning the impact of such changes is needed. The responsibilities of the individual, the employer and the government to ensure the optimal balance between these duties, should also be understood and recognised.

These new or revised ways of balancing the duty to treat with the duty to protect healthcare workers from infections is important to focus on before the next pandemic, to give both patients and healthcare personnel the care they need.

## Data Availability

The datasets for this study are not publicly available due to legal and ethical restrictions (the participants of this study did not agree for their data to be shared publicly), but they are available from the corresponding author on reasonable request.

## Abbreviations

AIDS: Acquired Immuno Deficiency Syndrome
HIV: Human Immuno-deficiency Virus
PPE: Personal Protective Equipment
SARS: Severe Acute Respiratory Syndrome
